# Internal Morphology of Mandibular Second Premolars Using Micro-Computed Tomography

**DOI:** 10.3390/jimaging9120257

**Published:** 2023-11-23

**Authors:** Thomas Gerhard Wolf, Samuel Basmaci, Sven Schumann, Andrea Lisa Waber

**Affiliations:** 1 Department of Restorative, Preventive and Pediatric Dentistry, School of Dental Medicine, University of Bern, CH-3010 Bern, Switzerland; samuel.basmaci@students.unibe.ch (S.B.);; 2 Department of Periodontology and Operative Dentistry, University Medical Center of the Johannes Gutenberg-University Mainz, DE-55131 Mainz, Germany; 3 Institute of Anatomy, University Medical Center of the Johannes Gutenberg-University, DE-55128 Mainz, Germany; sven.schumann@uni-mainz.de

**Keywords:** internal morphology, mandibular second premolar, micro-CT, number of canals, root canal configuration

## Abstract

To examine root canal morphology of mandibular second premolars (Mn2P) of a mixed Swiss-German population by means of micro-computed tomography (micro-CT). Root canal configuration (RCC) of 102 Mn2P were investigated using micro-CT unit (*µ*CT 40; SCANCO Medical AG, Brüttisellen, Switzerland) with 3D software imaging (VGStudio Max 2.2; Volume Graphics GmbH, Heidelberg, Germany), described with a four-digit system code indicating the main root canal from coronal to apical thirds and the number of main foramina. A total of 12 different RCCs were detected. 1-1-1/1 (54.9%) was most frequently observed RCC, followed by 1-1-1/2 (14.7%), 1-1-2/2 (10.8%), 1-2-2/2 (4.9%), 1-1-3/3 (3.9%), 1-1-1/3 (2.9%), 2-1-1/1 (2.9%) and less frequently 1-1-2/3, 1-2-1/2, 2-1-2/2, 1-1-2/5, 1-1-1/4 with each 1.0%. No accessory foramina were present in 35.3%, one in 35.3%, two in 21.6%, three and four in 2.9%, and five in 2.0%. In 55.9% Mn2Ps, accessory root canals were present in apical third and 8.8% in middle third of a root. Connecting canals were observed less frequently (6.9%) in apical and 2.9% in the middle third, no accessory/connecting canals in coronal third. Every tenth tooth showed at least or more than three main foramina. Almost two thirds of the sample showed accessory root canals, predominantly in apical third. The mainly single-rooted sample of Mn2Ps showed less frequent morphological diversifications than Mn1Ps.

## 1. Introduction

The mandibular second premolar is usually described as a single-rooted tooth with mainly one main root canal. However, a wide variation of its internal morphology is described in the literature [1,2]. For successful nonsurgical and surgical treatment by general dental practitioners as well as specialists, it is important to locate all root canals to remove the pulp tissue or necrotic debris [3]. Incomplete removal of debridement of the root canal is the main reason for unsuccessful endodontic treatment, followed by incorrect obturation [4]. Although there are various methods to study root canal morphology, radiographic analysis, clearing and macroscopic sectioning techniques were commonly used in previous studies [5,6,7].

Since the development of X-ray computed tomography (CT) about five decades ago, increasingly advanced diagnostic imaging technologies have been developed for medical and dental practice and routine use for both clinical and research purposes. Images taken from different angles will produce three-dimensional spatial distribution maps of material density in developing tissues such as bone or teeth or materials [8]. Conventional radiography is limited to generating two-dimensional images, showing the summation of material attenuation along the X-ray path [8]. While the first micro-CT scanners developed were mostly custom-made and not widely used, today’s modern systems are available on the commercial market and are widely used in industry and academic research. Micro-CT devices offer the possibility of examining a wide range of samples such as teeth or bones as mineralized tissues as well as materials such as polymers or ceramics. As part of in vivo research, soft tissues such as gingiva or the lungs are now also visualized using contrast agents; this method is mainly used in small living animals such as mice [9].

High-resolution detectors and micro focal spot X-ray sources allow projections in micro-CT devices to be rotated through multiple viewing directions to produce reconstructed 3D images of specimens. The generated images represent spatial distribution maps of linear attenuation coefficients. They are characterized by energy of the X-ray source and the atomic composition of the material sample. The internal characteristics of the same sample can be examined many times in a standardized and reproducible manner and can be used for biological and mechanical testing due to the non-destructive nature of the imaging process. Its use is now standard in several academic fields [9,10].

### 1.1. Root Canal Morphology

There is great variation as well as complexity in internal root canal morphology with e.g., accessory root canals, isthmuses or junctions, apical delta, or multiple foramina. Good knowledge and a broad understanding of the possible and expected morphological and anatomical conditions in the three-dimensional root canal system are important for a dentist, especially an endodontist specializing in root canal treatment, to make the right treatment decisions, and, above all, to weigh up the choice of instruments and methods accordingly. Clinically, conventional two-dimensional X-rays are usually taken, and in rare cases cone beam computed tomography (CBCT) images of a very limited area are also taken. These X-rays are used for diagnostic imaging, whereby the two-dimensional X-ray technique using intraoral images is the gold standard and CBCT images are only used as a 3D technique in exceptional cases. Here, not only a strict indication, but also radiation protection and hygiene must be considered, in addition to the financial aspect of the usually very high costs, which often must be borne by the patient. For dental ex vivo research using micro-CT systems, a non-invasive and precise examination is possible, which generates a large amount of information that is also often irrelevant for clinical decision-making, as the areas depicted in the image may be visible, but still cannot be adequately instrumented, prepared, or reached with irrigation fluids using systems available on the market today, let alone sealed using cement or warm obturation methods [11]. Micro-CT enables the analysis of fine structures of the dental hard tissue to obtain both quantitative and qualitative results of the root canal system, such as the root canal or pulp chamber cavity [12]. In this way, morphological conditions, such as diameter or enamel and dentin thickness, can also be measured in different sections of the tooth, such as the crown or root [12].

### 1.2. Analysis of the Morphology of the Root Canal

Micro-CT can be used to analyze the structure of a tooth. Micro-CT has been widely used in dental research to obtain both qualitative and quantitative results for pulp cavity and root canal morphology studies [13]. It is possible to use the reconstructed images to measure the morphological characteristics of the pulp chamber, the volume ratio in the horn, floor, and total area of the pulp chamber as well as the diameters of the buccal and lingual openings of the root canals and to compare them between different groups [14]. Regarding the analysis of the root canal morphology, it is possible to calculate the volumes and surfaces of the individual root canals and to measure and subsequently evaluate both the root canal configuration and the diameter of the root canal at different measuring points independently of the model. Thus, the curvature of a root canal can also be measured by creating an imaginary central axis, calculating the rotational speed of the tangent vector at a specific point on the central axis and converting this speed into the curvature of the root canal using special mathematical modeling software [15,16]. The different parts of a tooth can then be reconstructed or visualized together in three dimensions [12].

### 1.3. Micro-CT Applications in Dental Research

The different parts of a tooth can be reconstructed in 3D, often making the tooth structure transparent, the pulp chamber and root canal system opaque and the pulp red [2]. Both the external and internal morphology of a tooth can be easily reconstructed, and the relationship between the external and internal macro-morphology of the complex crown and root can be analyzed [17]. The acquired micro-CT can therefore serve as a basis for further analysis of root canal anatomy in experimental endodontology, for preclinical training in basic endodontic procedures and for valuable mathematical modeling of tooth morphology [12].

In the last decade, CBCT has been the commonly used method to examine the root canal system of mandibular second premolars [2]. However, to the best of the authors’ knowledge, root canal morphology of Mn2P using micro-computed tomography (micro-CT) has not been reported to date. Micro-CT has been described as a non-invasive, non-destructive, high-resolution, and reproducible ex vivo method that, when combined with 3D software imaging, can be considered the most accurate method for studying root canal morphology [18], as well as the gold standard in ex vivo research on internal morphology of teeth [19]. With the advancement of modern three-dimensional imaging, a more precise description of root canal systems and configurations (RCC) [20,21] became necessary. While established root canal classification systems of Vertucci [22] and Weine et al. [23] are still widely used, they are limited in terms of precision when describing an individual root canal morphology, especially for complex root canal systems. For this reason, the method of Briseño Marroquín et al. [20] was used in addition to the commonly used root canal configurations [22,23]. A four-digit code is used to describe the internal morphology of the corresponding tooth, with the root described in thirds from apical to coronal and the fourth digit denoting the number of main foramina [20]. Comprehensive knowledge and understanding of the root canal anatomy and morphology form the basis for successful endodontic non-surgical as well as surgical root canal treatment. Based on this knowledge and experience, the clinical practitioner selects the necessary means and instruments for the choice of treatment, which should enable better treatment decisions to be made with the aid of the anatomical-morphological conditions obtained.

The aim of this study was to analyze the internal morphology of the root canal system of mandibular second premolars of a mixed Swiss-German population using micro-CT.

## 2. Materials and Methods

### 2.1. Teeth Selection

A total of 102 extracted mandibular second premolars (Mn2Ps) were collected from university medical centers and private practices in Switzerland and Germany for reasons not related to this study. Only teeth that were clearly identified as mandibular second premolars by three independent observers (A.L.W., S.B., T.G.W.) were included in this study according to the description of Scheid and Weiss [24]. Inclusion criteria were fully developed roots, no coronal or radicular resorption, no caries, no root fractures, and no previous endodontic treatment. Otherwise, the teeth were excluded. All teeth examined in this study were categorized as so-called excess material; no information on the reason for extraction could be provided. A sample size calculation of teeth that were extracted period of 6 months from 1 April to 31 October 2022, for reasons unrelated to this study that could be used for evaluation according to the above criteria was calculated using proportion test with a confidence level of 95% and an expected prevalence of 90%. To the sample size of *n* = 97, 5% teeth were added in case of unexpected artifacts that would have led to an impossible analysis or failure. The adherent soft tissue and calculus were removed by ultrasonic debridement (Pieton 150; EMS Dental, Nyon, Switzerland) and manual scalers. After this cleaning process, the specimens were stored in 2% chloramine solution (Sigma-Aldrich, St. Louis, MI, USA) until examination.

### 2.2. Morphological Analysis by Micro-Computed Tomography

Mandibular second premolars were scanned using a predetermined method [2,16,25] in a desktop micro-computed tomography unit (*µ*CT 40; SCANCO Medical AG, Brüttisellen, Switzerland) with settings of 70 kV and 114 mA, resulting in 800–1200 slices per tooth. To differentiate the tooth structures, the images obtained were visualized by displaying them in dummy colors in the 3D reconstructions of the micro-CT scans using specialized software (VGStudio Max 2.2; Volume Graphics GmbH, Heidelberg, Germany). The structure of the teeth was color-coded using the software as follows: the pulp chamber and root canal system were coded red, coronal enamel was coded white, and dentin was coded transparent gray. The root canal system in this study is classified according to the Briseño Marroquín et al. classification system [20], which describes the internal morphology with four digits by dividing the root into thirds. Each of the first three digits represents the number of root canals at the coronal border of the apical, middle, and coronal thirds. The fourth digit is separated by a slash and represents the number of main foramina at the apex. A main foramen is defined originates from the same canal and has a diameter of at least 0.2 mm [20]. The number of connective and accessory canals and the number of accessory foramina were also examined. A connecting canal was defined as that which connects a root canal to the same or another canal without merging into the periapical tissue. The results are expressed by absolute and relative values depending on the number of samples.

## 3. Results

**Figure 1:**
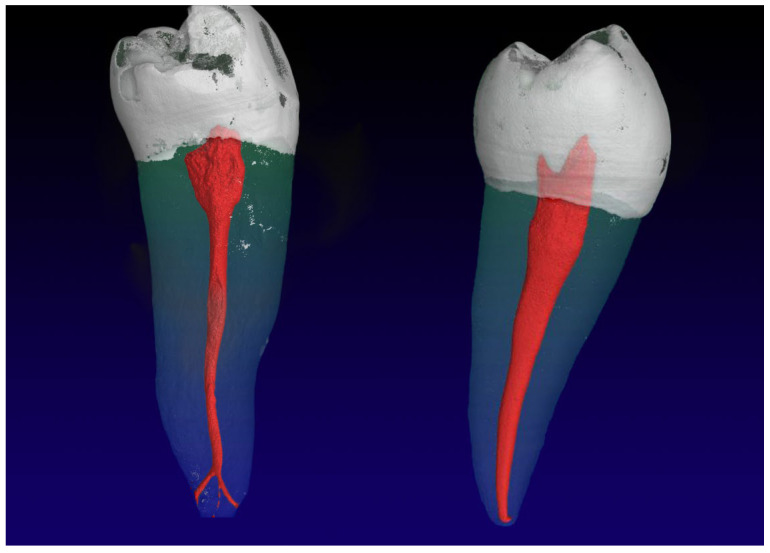
Mandibular second premolars with a 1-1-1/2 RCC (**left**) and an accessory root canal in the apical third of the root; and a 1-1-1/1 RCC (**right**) with an oval shaped canal.

**Figure 2:**
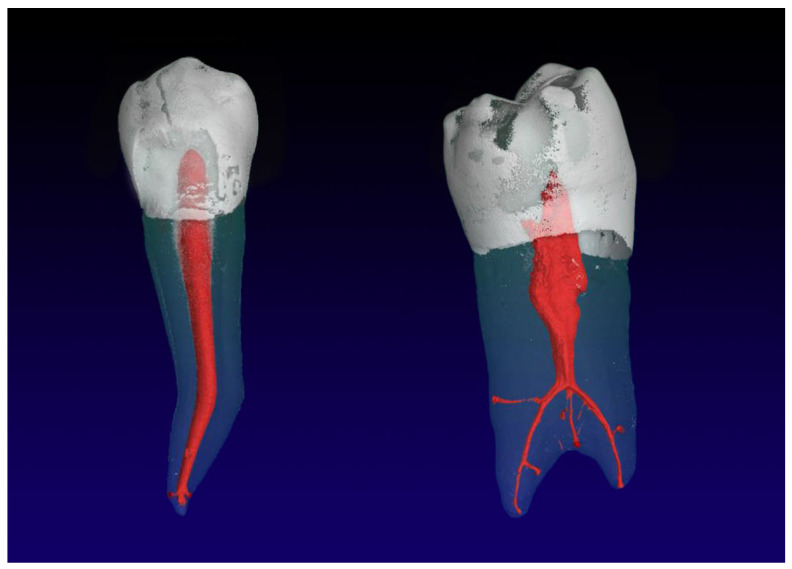
Mandibular second premolars with one root with a 1-1-1/3 RCC (**left**), and two roots (**right**) with a 1-1-2/2 RCC and five accessory root canals in the apical third of the root.

The sample of the Mn2Ps examined showed 100 (98.0%) single-rooted and two samples with two roots (2.0%). The cervical and middle parts of the root were fused in both teeth. 1-1-1/1 was the most frequently observed root canal configuration (RCC) (54.9%) (Figure 1), followed by 1-1-1/2 (14.7%). The RCCs of 1-1-2/2 (10.8%), 1-2-2/2 (4.9%), and 1-1-3/3 (3.9%) were also identified (Figure 2). The 1-1-1/3 and 2-1-1/1 RCCs were each present in 2.9% of the Mn2Ps studied. Five other RCCs were present in 1.0% of the Mn2Ps studied: 1-1-2/3, 1-2-1/2, 2-1-2/2, 1-1-2/5, 1-1-1/4 (Table 1).

The majority of Mn2Ps examined had one physiological foramen in 57.8% of the samples. Two physiological foramina were present in 32.4%, three in 7.8%, and four or five each in 1% (Table 2). No or one accessory foramen was present in 35.3% of the teeth examined, while two accessory foramina were present in 21.6% of the teeth examined. Three (2.9%), four (2.9%), and five (2.0%) accessory foramina were observed less frequently (Table 2).

The Mn2Ps showed no connecting canals in 90.2%. These canals occurred only in the middle (2.0%) and apical thirds (7.8%) of the root (Table 3). One sample showed two connecting canals and another tooth three connecting canals in the middle third, respectively. One tooth had two connecting canals in the apical third. Accessory root canals were present in 55.9% of the roots in the apical third and in 8.8% of the roots in the middle third, 35.3% of the samples had no accessory root canals (Table 3).

## 4. Discussion

The purpose of this study was to analyze the internal morphology of the root canal system of mandibular second premolars of a mixed Swiss-German population using micro-CT. Different techniques have been used to study root canal morphology such as sectioning, macroscopic sections, radiographs, and CBCT imaging [2,5,6,7,20,26]. Several previously commonly used methods, such as sectioning, are invasive and cannot visualize fine details of the root canal system compared to micro-CT. The loss of tooth structure (approx. 0.2 mm diamond strips) due to sectioning is even greater than the section thickness itself (approx. 0.145 mm), which is a clear disadvantage compared to e.g., radiography or CBCT imaging. The CBCT technique as non-invasive methodology allows ex vivo as well as in vivo examinations.

To the best of authors’ knowledge, this is the first study investigating mandibular second molars by means of micro-CT. Micro-CT, as a nondestructive, noninvasive, reproducible, and high-resolution ex vivo method, allows visualization of fine structures and is now described as the most precise method for the study of root canal morphology [8,9,27]. It is therefore not surprising that modern three-dimensional imaging has replaced sectioning techniques as well as conventional two-dimensional radiography for anatomical studies [28]. Recent studies investigating the morphology of mandibular second premolars have often been performed using CBCT with relatively large sample sizes [26,28]. CBCT seems to be a suitable method to analyze root canal morphology, although it does not show the high-resolution details of micro-CT [29].

The root canal configuration (RCC) systems of Vertucci [22] and Weine et al. [23] are widely used to describe the internal root morphology. However, various computerized imaging techniques, such as micro-computed tomography, have succeeded in imaging other RCCs that cannot be classified by the above classification systems. Therefore, in the present study, an elaborate four-digit classification system has been used, based on the division of the root into thirds and the use of the fourth digit to describe the number of main apical foramina [20].

While the Ma2P is typically described as single-rooted with a single canal, numerous case reports indicate several variations of anatomy and RCCs such as a triple-rooted tooth with three canals [30] or a single-rooted tooth with five canals [31]. However, these are individual cases; two or more rooted teeth have rarely been reported [1,32]. In the present study, the 1-1-1/1 RCC of Ma2Ps was the most frequently observed RCC with slightly more than half of the examined samples (54.9%). Similar findings were shown in the literature of Sert and Bayirli with 57% [33] using staining and clearing or 55.3% in males and 57% in females using CBCT [34]. The only directly comparable study of a German sample showed different data with 39.0% of 1-1-1/1 and 57.1% of 1-1-2/2 using CBCT [28]; combined RCCs of 1-1-1/2, 1-1-2/2, and 1-2-2/2 in the present study 30.4%. In the literature, data are also found in earlier studies such as in a Mexican sample with 98.8% by radiography [5]. Although the 1-1-1/1 RCC is the most common in the current report, it is represented in a rather low percentage and thus strongly disagrees compared to a recently published systematic review reporting frequencies of CBCT studies up to 99.6% in mandibular second premolars [2]. Reasons for the differences may include methodological differences in the study, the classification used, ethnic, gender, or sample size differences [2].

### 4.1. Methodology

Various methods can be used to identify and document the root canal system [12]. It is important to select the appropriate method based on the desired criteria. Many of these techniques were used early on, but their limitations have always been debated and the search for new methods has continued. The most used methods for analyzing root morphology are radiographs, radiography with a contrast agent, staining and clearing, staining and clearing with electron microscope scanning, sectioning, CBCT, and micro-CT, whereby the last two mentioned are the most frequently used methods in recent studies [12].

#### 4.1.1. Cone Beam Computed Tomography (CBCT)

CBCT uses an extraoral, cone-shaped X-ray beam that rotates around the object to be imaged. This allows the internal morphology of the object—in this case the complex root canal system—to be visualized and observed and produces a 3D volume from a high number of projection images, which is a decisive advantage. In contrast with conventional CT, a lower radiation dose is applied. Compared to the digital 2D X-ray image, CBCT allows a more precise determination of the anatomy of the root canal system. Furthermore, CBCT can be used both for in vivo and ex vivo research, is less invasive and can be used as an aid in diagnosis, preoperative assessment, and during treatment. Metal restorations, implants, and sometimes root fillings cause artifacts. The exposure time for CBCT is significantly longer than for conventional images. Even the slightest movement could result in an image with minimal diagnostic use. If a large voxel size is selected, fine structures such as a broken instrument or a thin canal may not be visible [35,36,37].

Abdinian et al. conducted a study in which they compared CBCT with digital radiography in the detection of vertical root fractures [38]. Since the diagnosis of root fracture is challenging, they set themselves the goal of finding a suitable diagnostic tool. They realized that the sensitivity (correct detection of root fractures) of a CBCT is higher compared to a digital X-ray, but the specificity (correct detection of non-fractured roots) is lower. Their recommendation is to first X-ray the teeth from three different angles (mesial eccentric, orthognathic, distal eccentric) and use a surgical microscope before considering a CBCT in addition to providing a justifying indication [38].

Matherne et al. showed in their study that radiographs fail to reveal at least one canal in 40% of cases compared to CBCT [37]. However, CBCT can also be used to investigate the curvature of root canals and is recognized as a suitable imaging technique tool [37,39]. It should also be noted that the expected radiation dose for CBCT imaging is 0.02 mSv, compared to 0.001–0.006 mSv for single tooth imaging [40].

#### 4.1.2. Micro-Computed Tomography (Micro-CT)

Micro-computed tomography (micro-CT) can be used to analyze different parts of the tooth and examine the morphology. In addition to the morphology of root canals, it is also possible to examine the frequency of different root canal systems. A three-dimensional software then allows the teeth to be viewed in a 3D coordinate system and searched according to the desired criteria. This technique enables a non-destructive, quantitative analysis of selected variables such as enamel density and root canals. Micro-CT is increasingly used due to its high resolution, accuracy, and detailed quantitative and qualitative measurements [41,42]. For this purpose, the teeth are scanned with an isotopic resolution of 20 µm in a desktop micro-CT unit. The necessary settings (e.g., 70 kV and 114 mA) are used to generate a certain number of slices per tooth. With these modules, the tooth structures are visualized by the 3D reconstruction of the micro-CT scan. The visualization is done by color coding: the pulp chamber and the root canal system in red, the enamel in white, and the dentin in grey [2,20,43,44].

Various software programs can be used to evaluate the data. These software programs can be used to analyze and measure 3D objects. The selected tooth is displayed in four windows. Three windows show the transverse and longitudinal sections through the molar. The fourth window shows the entire molar for orientation. In addition, the tooth is displayed in a Cartesian coordinate system, whereby the object can be examined in different axes. Each axis shows the height of the tooth in millimeters [mm]. Thanks to this information, the position of the canal or its best section can be quickly localized.

### 4.2. Limitations

Several limitations should be mentioned. While numerous older studies [2] have no or small sample sizes, most studies in the nearer past, especially when using CBCT in in vivo studies, show higher sample sizes. The present study used a comparatively smaller sample size than many recent studies; however, this study is the first micro-CT study, and a prior statistical sample size calculation was performed. Another issue is the technical nature of the device and imaging. There are currently several micro-CT devices and software solutions available on the commercial market for the analysis of micro-CT data for dental ex vivo research. In the present study, a commonly used and already-established methodology used by different research groups in laboratory studies was employed. The chosen settings of hardware and software are based on both experience values and previously established settings that have already been performed in standardized and reproducible analyses [16,18,20,25,43,44,45,46]. However, alternative combinations of software and hardware are also possible, which most likely offer similar technical solutions and should provide the same results according to standardized, reproducible principles. Precise imaging of the fine structures of root canal morphology allows observers to achieve unambiguous descriptions of internal morphology and possible configurations. Even though a possible bias can be reduced by using multiple observers, a subjective influence cannot be excluded when describing the methodology used. The methodologies used according to Briseño-Marroquín et al. [20], Vertucci [21], and Weine et al. [23] certainly allow numerous and detailed descriptions, but also have limitations. However, methods for describing intern root canal morphology such as Ahmed and Dummer [21] offer even more detailed, but at the same time more complex descriptions of the internal root canal morphology.

The current study also showed that connecting or accessory canals may be present in many cases, especially in the apical, but also in the middle third of the root. These structures cannot be reached by mechanical treatment with files, which is why chemical disinfection with root canal irrigation solutions is essential. This fact underlines the importance of combined mechanical and chemical root canal disinfection, as well as necessary sufficient obturation for successful root canal treatment.

## 5. Conclusions

Within the limitations of the current study several conclusions can be drawn:

- The most frequently root canal configurations (RCC) observed mandibular second premolar (Mn2P) were 1-1-1/1 (54.9%), 1-1-1/2 (14.7%), 1-1-2/2 (10.8%), and another nine RCCs.
- The majority of Mn2Ps were single-rooted (98%) and presented with one (57.8%) or two physiological foramina (32.4%), almost one in ten teeth showed at least three main foramina.
- Almost two thirds showed accessory root canals, predominantly located in the apical third.
- The mainly single-rooted sample of Mn2Ps showed less frequent morphological diversifications than Mn1Ps.

## Figures and Tables

**Table 1:** Absolute (*n*) and relative (%) results of the examined sample of mandibular second premolars using micro-CT, described with the methods of Weine et al. [23], Vertucci [21], and Briseño Marroquín et al. [20] (*n* = 102)**.**

Weine et al., 1969 [23]	Vertucci, 1984 [21]	Briseño Marroquín et al., 2015 [20]	Absolute (*n*)	Mean (%)
I	I	1-1-1/1	56	54.9
	V	1-1-1/2	15	14.7
		1-1-1/3	3	2.9
	V	1-1-2/2	11	10.8
		1-1-2/3	1	1.0
	V	1-2-2/2	5	4.9
	VII	1-2-1/2	1	1.0
	II	2-1-1/1	3	2.9
	VI	2-1-2/2	1	1.0
		1-1-3/3	4	3.9
		1-1-2/5	1	1.0
		1-1-1/4	1	1.0

**Table 2:** Absolute (*n*) and mean (%) frequency of the physiological and accessory foramina observed in the coronal (Co), middle (Mi) and apical (Ap) thirds of mandibular second premolars (*n* = 102).

Foramina		
**Main Apical Foramina**	***n***	**%**
/1	59	57.8
/2	33	32.4
/3	8	7.8
/4	1	1.0
/5	1	1.0
**Accessory Foramina**	***n***	**%**
0	36	35.3
1	36	35.3
2	22	21.6
3	3	2.9
4	3	2.9
5	2	2.0

**Table 3:** Absolute (*n*) and mean (%) frequency of connecting and accessory canals observed in the coronal (Co), middle (Mi) and apical (Ap) thirds of mandibular second premolars (*n* = 102).

Canals		
**Connecting Canals**	***n***	**%**
None	92	90.2
Co	0	0.0
Mi	3	2.9
Ap	7	6.9
**Accessory Canals**	***n***	**%**
None	36	35.3
Co	0	0.0
Mi	9	8.8
Api	57	55.9

## Data Availability

Data are contained within the article.
